# Co-encapsulation of vitamin D and rutin in chitosan-zein microparticles

**DOI:** 10.1007/s11694-022-01340-2

**Published:** 2022-02-11

**Authors:** Fideline Laure Tchuenbou-Magaia, Roberta Tolve, Uchechukwu Anyadike, Marco Giarola, Fabio Favati

**Affiliations:** 1 School of Engineering, Division of Chemical Engineering, University of Wolverhampton, Wolverhampton, WV1 1LY UK; 2 Department of Biotechnology, University of Verona, Strada Le Grazie 15, 37134 Verona, Italy; 3 Centro Piattaforme Tecnologiche (CPT), University of Verona, 37134 Verona, Italy

**Keywords:** Anti-solvent precipitation, Co-encapsulation, Chitosan, Rutin, Vitamin D_3_, Zein protein

## Abstract

There is a growing interest in co-encapsulating multiple species to harness potential synergy between them, enhance their stability and efficacy in various products. The aim of this work was to co-encapsulate vitamin D_3_ and rutin inside chitosan-zein microparticles using a simple and easily scalable process for food fortification. This was achieved via anti-solvent precipitation coupled with spray-drying. Free-flowing powders of spherical microparticles with wrinkled surface and particle size < 10 μm were obtained. The encapsulation efficiency was 75% for vitamin D_3_ and 44% for rutin and this could be attributed to their different molecular size and affinity to the aqueous phase. The physicochemical properties were characterized by X-Ray powder diffraction and Fourier transform infrared spectroscopy. The two crystalline bioactive compounds were present in the microparticles in amorphous form, which would allow for better bioavailability when compared to non-encapsulated crystalline solid. Therefore, the obtained microparticles would be suitable for use as food ingredient for vitamin D_3_ fortification, with the co-encapsulated rutin acting as stability and activity enhancer.

## Introduction

Vitamin D is a lipophilic molecule crucial for calcium and phosphorus homeostasis, bone mineralization and also needful for preventing and protecting against a variety of non-skeletal disorders, such as cardiovascular disease, obesity, glucose metabolism, mood disorders, muscular function, tuberculosis, and colorectal adenomas [1]. Moreover, several studies evidenced the link between vitamin D deficiency and an increased risk of respiratory tract infections, influenza [2] increase in the duration of hospital stay, disease harshness, and greater mortality risk for COVID-19 patients [3]. This important role of vitamin D is attributed to its implication in the modulation and regulation of the immune and oxidative response alongside its direct antiviral effects against enveloped viruses such as coronavirus [4]. It is therefore apparent that sufficient vitamin D is crucial not only for the overall health but could play a significant role in the fight against COVID-19 and its devastating consequences as demonstrated by Grant et al. [5] which suggested vitamin D supplementation to reduce the risk of influenza and COVID-19 infections and deaths. However, vitamin D deficiency is found in about 80% of the population, which is mainly attributed to insufficient sunlight exposure and insufficient dietary intake as only limited foods such as eggs, liver, mushrooms, and oily fish contain this vitamin [6, 7].

**Fig. 1:**
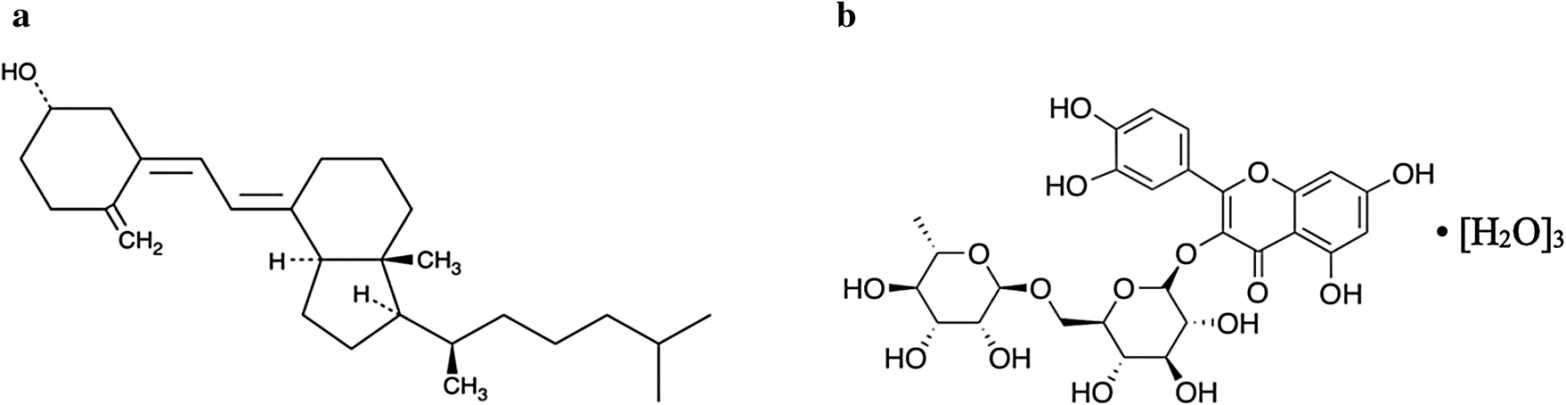
Vitamin D_3_ (**a**) and rutin hydrate structure (**b**)

Vitamin D is a mixture of steroid derivatives and the two most important forms are vitamin D_2_-ergocalciferol, present in plant-based foods, and vitamin D_3_-cholecalciferol, present in animal-based foods. Furthermore, the biologically active form of vitamin D, vitamin D_3_ (Fig. 1a) is synthesized in the skin by exposure to sunlight, hence its nickname the "sunshine vitamin” [8]. The dietary reference value (DRV) for vitamin D is generally between 8.5 and 20 μg per day according to age, different physiological states (pregnancy, lactation) and different health authority outlook [7]. Despite the relatively low DRV, the high number of people with deficiency in vitamin D, and now certainly exacerbated by the stay-at-home coronavirus measures, evidence the urgent need for vitamin D food fortification and supplementation, especially for the population who are at high risk of deficiency, such as older adults and people with dark skin [9]. Indeed, Public Health England [10] recommended vitamin D supplement containing 10 µg of vitamin D during autumn and winter for people not eating enough foods that naturally contain vitamin D or vitamin D fortified foods. On the other hand, vitamin D fortification is mandatory in Canada for milk and margarine since the 1970s and other foods such as fruit juice, soy milk and breakfast cereal have recently been added to the list [11], whereas this fortification is optional in the United States for milk, breakfast cereals, and calcium-fortified fruit juices [12]. The challenge of incorporating vitamin D into food lies in its high sensitivity to isomerization and oxidation when exposed to heat, light, moisture, or oxygen. This may cause a reduction in vitamin D concentration of the fortified products during processing and storage [13] whilst negatively affecting its physiological and health benefit [14] as well as the product sensory attributes. This challenge could be overcome through vitamin D encapsulation in appropriate protective matrices [15].

Simultaneous encapsulation of multiple bioactive compounds to develop a co-delivery system is common in the pharmaceutical area since it offers several health benefits and can bring potential synergistic effects [16]. This strategy is gaining attention in recent years also in food applications with examples of co-encapsulation of probiotics and green tea extracts [17], probiotics and omega-3 fatty acids [18], omega-3 fatty acids, phytosterol and limonene [19], coenzyme Q10 and vitamin E [20] and vitamins B12 and C [21]. In the present work, it was hypothesized that co-encapsulation of vitamin D with anti-oxidant molecules could enhance its biological function while improving its stability as recently reported by Scuto et al. [22] summarizing the chemopreventive potential of plant polyphenols and vitamin D through the activation of cellular redox response.

Rutin, known as quercetin 3-rutinoside or vitamin P (Fig. 1b), is a quercetin glycoside abundant in plants and fruit and well known for its scavenging properties of oxygen reactive species whilst being non-oxidizable when compared to other flavonoids such as myricetin and quercetagetin which may act as prooxidants [23]. Furthermore, rutin shows broad pharmacological activities, including anti-inflammatory and vasoactive effects, enhancement of antibiotics efficacy [24] and therapeutic effect for maintaining bone health and management of osteoporosis [25].

Different types of shell materials can be used to encapsulate bioactive compounds. Among them, the self-assembly property of zein protein has been investigated for the encapsulation of lipophilic components such as lipids, liposoluble vitamins, food colorants, flavours, anti-microbial agents, and antioxidants [26]. Zein protein is present in corn seeds and belongs to the prolamin group characterized by the presence of both lipophilic (3/4) and hydrophilic (1/4) amino acid residues. This particular feature makes zein soluble in mixtures of water and alcohol, and represent the basis of the protein’s ability to generate films and to self-assemble into microparticles. Up-to-date, encapsulation of a single compound using zein protein has been successfully developed for the nanoencapsulation of fish oil, essential oils and also vitamin D [27]. In recent years, some research has shown the synergistic effects of the co-encapsulation of more than one bioactive compound which would significantly enhance their bioactivity and functionality when compared to the encapsulation of a single component [28]. In this perspective, this work aimed to investigate whether vitamin D_3_ could be co-encapsulated with rutin in zein microparticles coated with chitosan using an easily scalable process for mass production. Chitosan is a natural polymer widely used in encapsulation due to its versatility and film-forming properties. It was anticipated that the chitosan outer layer may provide a protective layer that would overcome the limitation of zein as single shell material due to its brittle nature, which makes it sensitive to failure and rapid crack propagation under stresses, such as those encounters during mixing and incorporation of the encapsulated microparticles into food products.

## Materials and methods

### Materials

Vitamin D_3_ cholecalciferol (≥ 99.8% purity), rutin hydrate (≥ 94% purity), zein protein and chitosan low molecular weight and other chemicals were purchased from Sigma (Sigma-Aldrich, Merck Sigma, Italy). Ultrapure water was used for analysis whereas distilled water was used for the preparation of microparticles. All the chemicals were of analytical grade.

### Production of microparticles

**Fig. 2:**
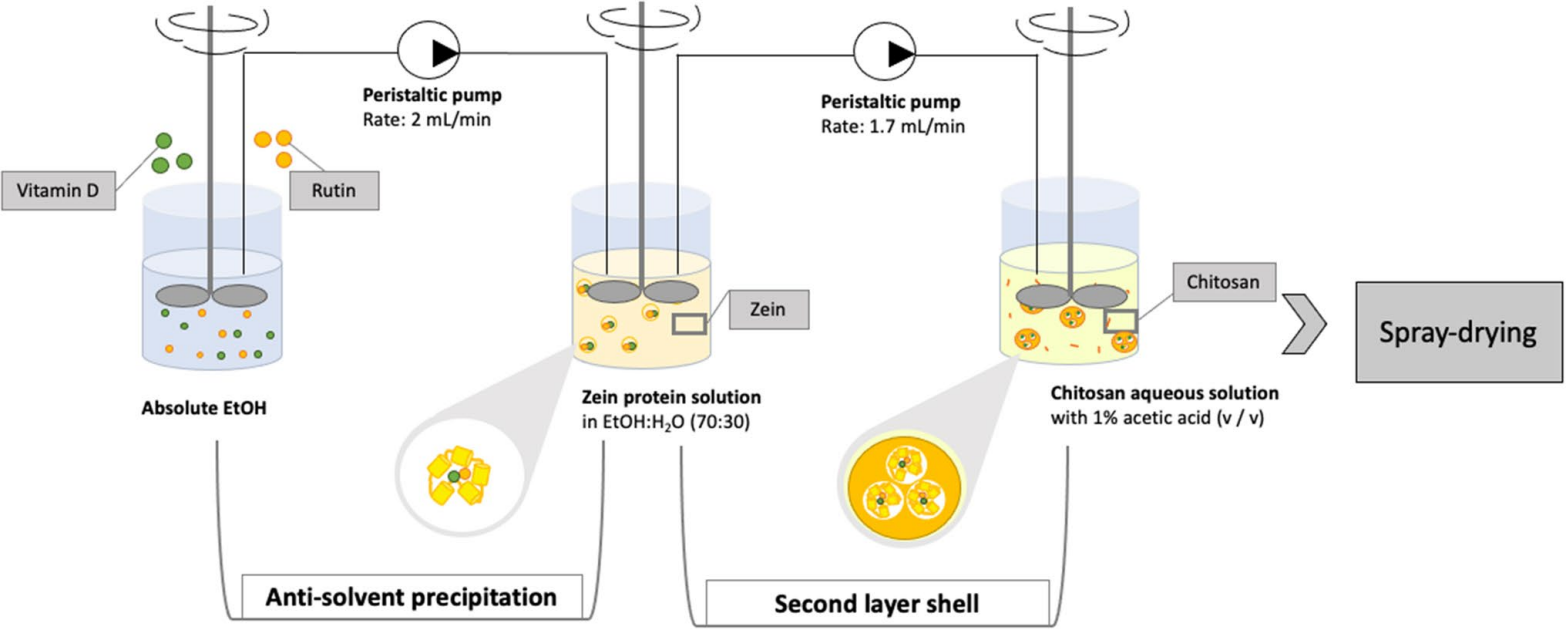
Graphical description of the used procedure

According to the available data in the literature [27] and on the base of preliminary experiments, Chitosan-Zein coated microparticles containing vitamin D_3_ and Rutin and (CZ-VDR), were prepared using a combination of anti-solvent precipitation and different drying using a freeze-dryer, oven-dryer and a spray-dryer. CZ-VDR microparticles were produced as follows: 45 mg of vitamin D_3_ and 30 mg of rutin were dissolved in 15 mL of absolute ethanol and then pumped (2 mL/min) into 40 mL of a zein solution prepared by dissolving 0.5 g of zein powder in 40 mL of ethanol–water (70% v/v) under stirring (Heavy stirrer, ALC International Srl Class I, Italy) for one hour. The resultant mixture was then pumped (1.7 mL/min) using a peristaltic pump (Gilson Minipuls 3, Villier le Bel, France) into 150 mL of a chitosan solution prepared by dissolving 0.5 g of chitosan in 150 mL of deionized water added with acetic acid (1% v/v). The pumping system was set so to keep the tip of the outlet tube about 3 mm above the solution of chitosan and, during the pumping, the pH of the chitosan solution was brought to 4 using a solution of 1 M sodium hydroxide in order to maintain the chitosan positively charged and the zein protein negatively charged. The mixture was maintained under the mixing condition of 200 rpm for one hour (Fig. 2). Control samples with only rutin (CZ-R) and without vitamin D_3_ and rutin (CZ) were also prepared. Microparticles suspensions obtained from this production step were subject to different drying techniques: freeze-drying, oven-drying and spray-drying. For freeze-drying, the suspension of microparticles was immediately frozen at − 80 °C and freeze-dried (ScanVac CoolSafe 90–80; LaboGene ApS, Lynge, Denmark) whereas other samples were dried by placing them overnight in an oven (M60-VN, MPM Instruments Srl, Bernareggio, Italy) at 50 °C or using a benchtop spray drier (Mini Spray Dryer B-290 Advanced, Büchi, Switzerland) operating at the following conditions: inlet temperature 180 °C, outlet temperature 85–90 °C; pump ratio 15% and aspirator ratio 95%. The microparticles were collected in containers sealed with screw caps until further use and analysis.

### Microparticles characterization

#### Morphology and size of microcaparticles

The morphology of the microparticles suspensions before and after chitosan coating was visualized under a Zeiss Axiovert 135 light microscope (Carl Zeiss S.p.A., Arese, Italy) at 100 X magnification and the ImageJ software (version 1.53a, National Institutes of Health, Bethesda, Maryland, USA) was used to set the scale-bar. Dried microparticles were subjected to analysis by scanning electron microscopy (SEM) to evaluate their morphology and size. For this purpose, sample CZ-VDR, CZ-R and CZ were fixed to stubs with colloidal silver and coated with gold through the MED 010 coater (Balzers, Milan) and, subsequently, examined under an electron scanning microscope (FEI Company, Eindhoven, Netherlands).

#### Encapsulation yield, loading capacity and encapsulation efficiency

The encapsulation yield (EY) was determined as the ratio of the amount of powder collected after every drying experiment to the initial quantity of solids contained in the feed suspension [Eq. (1)]:

1 $$EY \left( \% \right) = \frac{Mass\, of\, powder \,collected}{{Mass \,of\, solid \,fed }} \times 100$$

Quantification of vitamin D_3_ and rutin encapsulated via spray-drying was performed after cold extraction of the microparticles using a slight modification of the method described by Das et al. [29]. Specifically, 10 mg of microparticles were placed inside a mortar, sited in a box containing ice, and the bioactive compounds were extracted by adding drop by drop 10 mL of a cold 1% acetic acid–ethanol solution. Afterward the mortar was carefully covered with aluminium foil and the mixture was mixed for 30 s every 5 min, for a total extraction time of 60 min. The obtained mixture was then filtered using a 0.22 µm syringe filter. After discarding the first 3 mL, the filtrate was analyzed by HPLC for its content in vitamin D_3_ and rutin using a chromatography system (Gold 126 Solvent Module, Beckman, Cassina de' Pecchi, Milan, Italy) equipped with an autosampler (System Gold 508, Beckman) and a diode array detector (System Gold 168, Beckman). Detection and quantification of vitamin D_3_ and rutin were carried out a 264 and 258 nm, respectively. Separation of the moieties of interest was achieved by means of a C18 column (Synergi™ Fusion RP80, Phenomenex, Torrance, CA, USA) (4 μm, 250 × 4.60 mm) using as mobile phase acetonitrile:methanol (98:2 v/v) for vitamin D_3_ and water acidified with acetic acid (1% v/v): acetonitrile (75:25) for rutin. For analysis, 25 μL of the sample were injected onto the column and the elution was carried out working in isocratic mode with the mobile phase flow rate was set at 1 mL/min. Identification and quantification of vitamin D_3_ and rutin were achieved using the external standard technique, checking the linear response of the detector over the concentration range 1.56-50 mg/L for vitamin D_3_ and 1.56-100 mg/L for rutin. Once the content in vitamin D_3_ and rutin in the microparticles was assessed, it was possible to calculate the loading capacity (LC) and the encapsulation efficiency (EE) using Eq. (2) and Eq. (3) as reported by Huang et al. [30]:

2 $$LC \left( \% \right) = \frac{Bioactive\, compound\, in\, microparticle}{{Mass\, of \,microparticle}} \times 100$$

3 $$EE \left( \% \right) = \frac{Bioactive\, compound\, in\, microparticle}{{Mass \,of\, compound \,added \,to\, the\, formulation}} \times 100$$

The water activity (a_w_) of the spray dried microparticles was measured at 25 °C by using a HygroPalm instrument equipped with an HC2-AW sensor (Rotronic Italia Srl, Milano, Italy). The moisture content was assessed according to AOAC (method 44–15) [31].

#### X-ray powder diffraction and IR spectroscopy measurement

X-ray powder diffraction (XRPD) measurements were performed with a Thermo ARL X'TRA powder diffractometer (JASCO Europe srl, Cremella, LC, Italy) in Bragg–Brentano geometry, equipped with an X-ray source with a copper anode (Kα λ = 1.5405 Å) and a Si Peltier solid state detector (Li). The powder samples were ground in a mortar and then deposited in low-background sample holder for the data collection. The diffraction pattern was acquired from 5° to 90° *2θ* range with a resolution of 2834 points (step size 0.03 degrees) and a scan rate of 2.5°/min. The chemical analysis of rutin, vitamin D_3_ and the microparticles (CZ-VDR, CZ-R and CZ) was studied using the absorbed infrared radiation (FT-IR). The powder samples were incorporated into KBr pellets (1–3% by weight) and analyzed at room temperature using a Fourier-transform infrared spectrophotometer (FT/IR-660 plus, JASCO, Easton, MD, USA). This instrument allows to acquire the measurements in vacuum, obtaining excellent quality spectra and avoiding the presence of air and water during the measurement. Absorbance (A = − log (I/I0)) was obtained by measuring I, signal strength of the sample incorporated in KBr pellets and I0, signal strength of KBr. For each spectrum, 256 scans were carried out, in the medium infrared spectral range (MIR 4000–400 cm^−1^) with a resolution of 2 cm^−1^. Origin 2017 software (OriginLab Co., Northampton, MA, USA) was used for background subtraction and initial analysis of the collected IR spectra. The intensity of the spectra was shifted for better visualization and comparison between them.

### Statistical analysis

All the analyses were carried out in triplicate and the pertinent data are reported as mean value ± standard deviation. Comparison of means was conducted using the analysis of variance (ANOVA) with Post Hoc Tukey’s test at p < 0.05. Statistical analyses were performed by using the XLSTAT software (Ver 2020.5, Addinsoft SARL, Paris, France).

## Results and discussion

### Morphology and size of microparticles

**Fig. 3:**
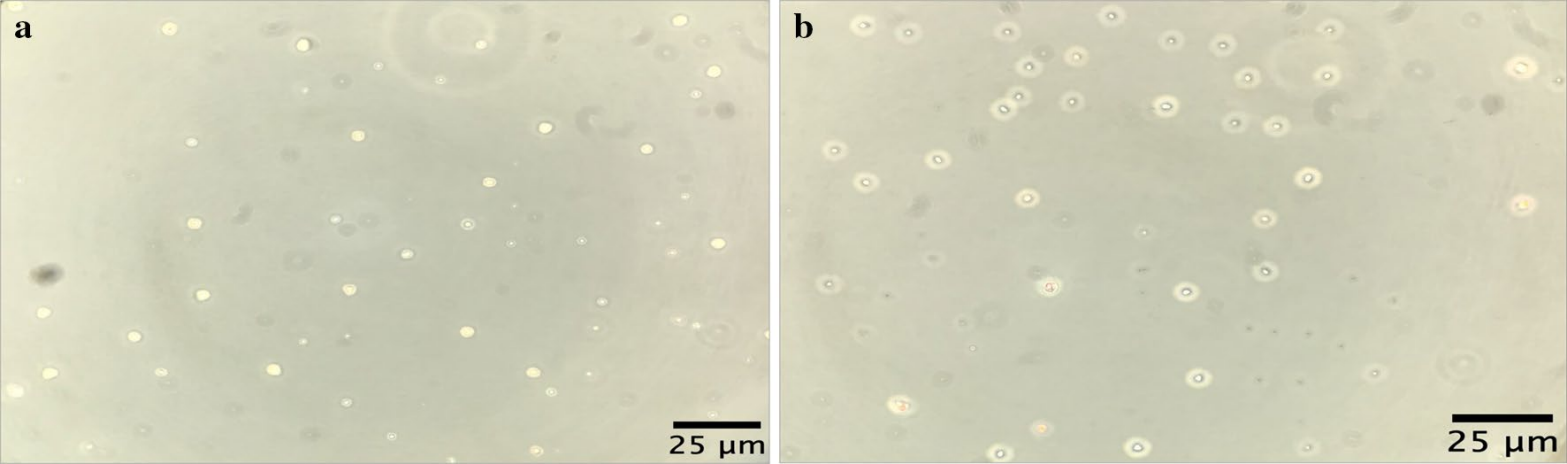
Optical microscope images of microparticles without any active compound made with zein (**a**) and zein + chitosan (**b**) as shell material

**Fig. 4:**
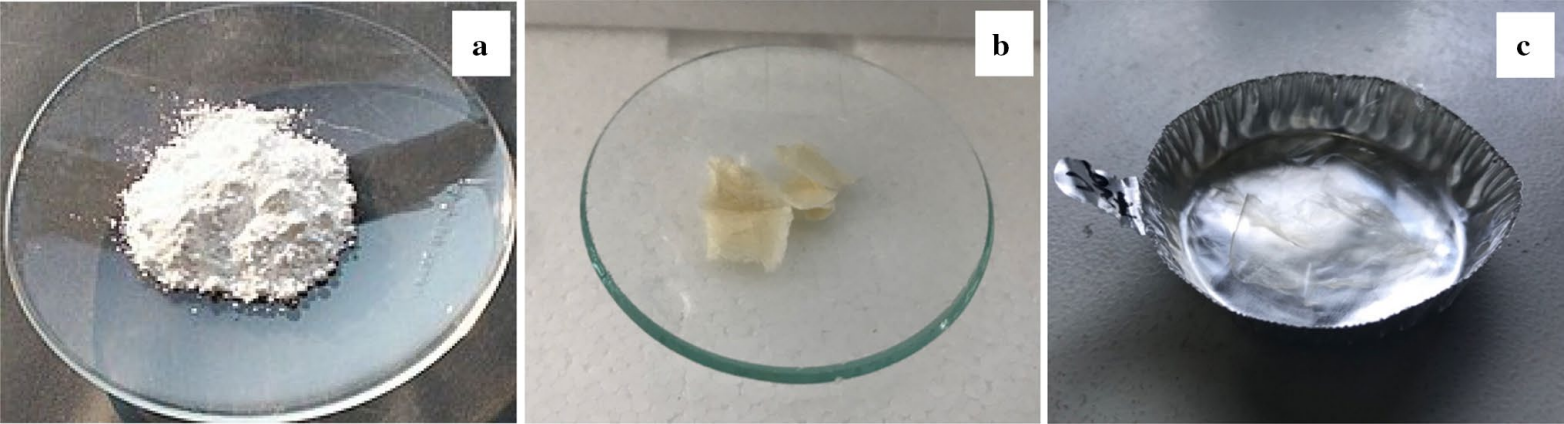
Dried Chitosan–zein microparticles with no active compound (CZ) obtained using different techniques: spray-drying (**a**) freeze-drying (**b**) and oven-drying (**c**)

**Fig. 5:**
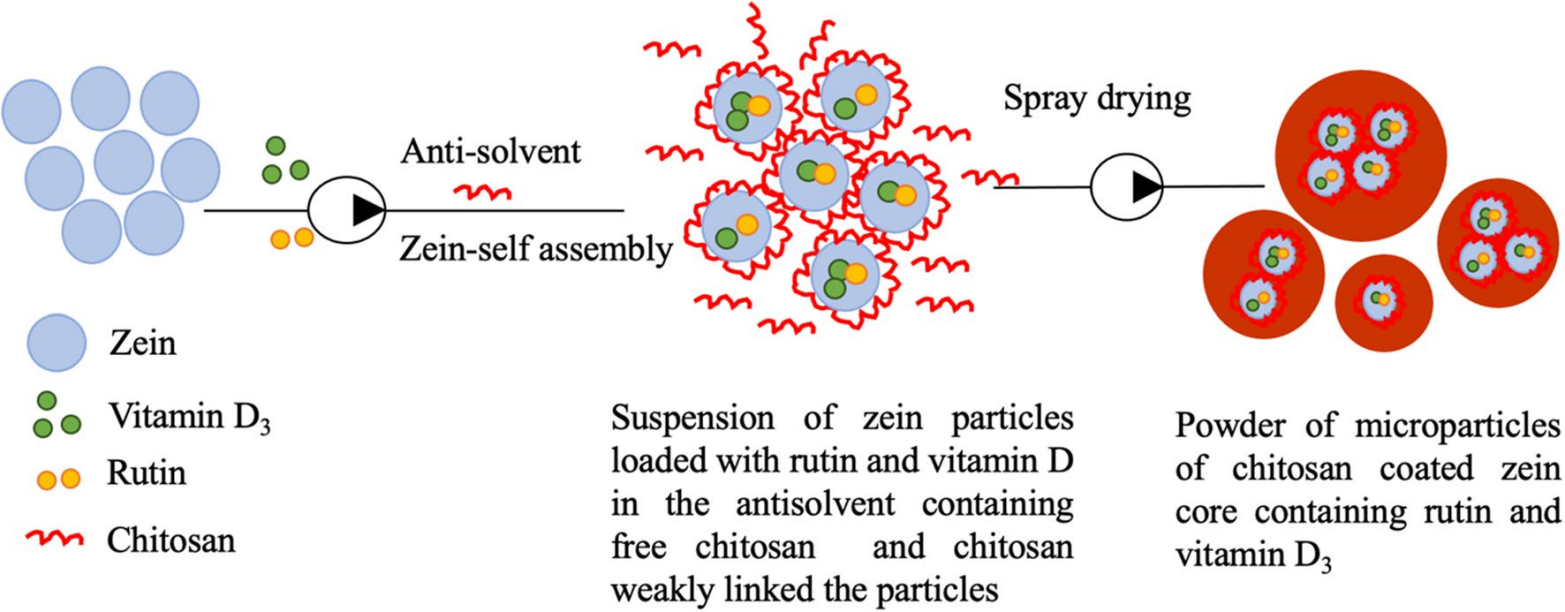
Schematic representation of the formation of chitosan-zein microparticles loaded with rutin and vitamin D_3_

**Fig. 6:**
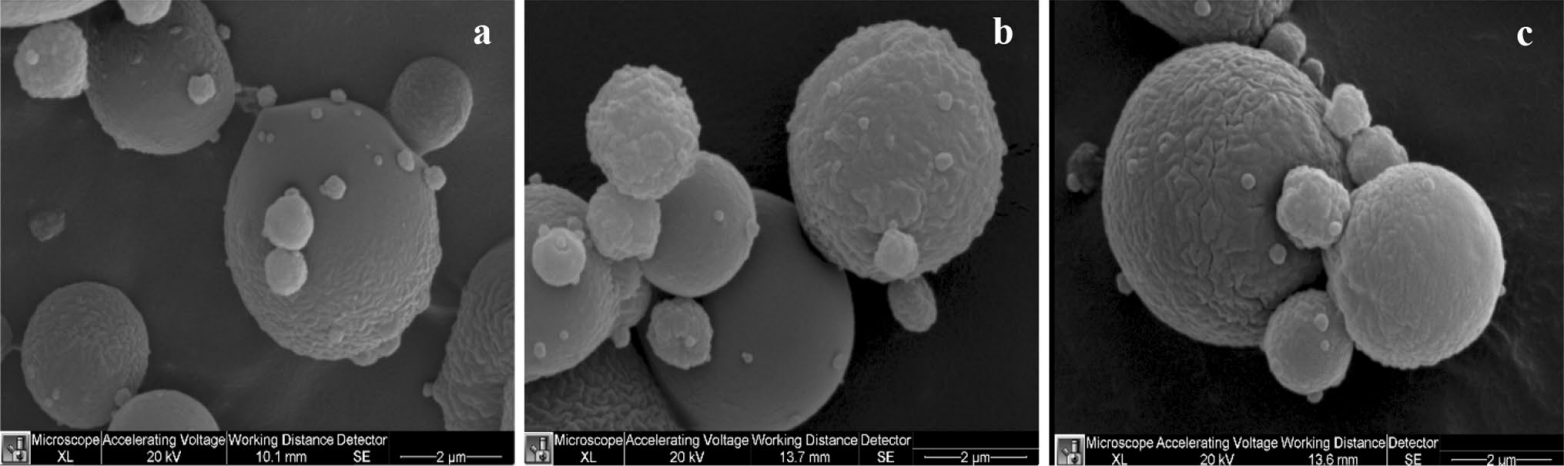
Scanning electron microscope image of microparticle (5000 x): **a** CZ (chitosan zein microparticles with no active compound), **b** CZ-R (chitosan-zein microparticles loaded with rutin), **c** CZ-VDR (chitosan zein microparticles loaded with vitamin D_3_ and rutin). The white bar in each image represents 2 μm

Double shell chitosan-zein microparticles were produced using the antisolvent precipitation method where zein particles were formed by continuously adding an aqueous-ethanol solution of zein into an aqueous chitosan solution (Fig. 3b) acting as anti-solvent, which resulted in transient supersaturation of zein and particles formation due to rapid ethanol diffusion, followed by drying. Chitosan-zein microparticles showed a particle size below 10 μm which was larger than the zein particles obtained by using only water as anti-solvent (Fig. 3a) as expected. These results are in agreement with the literature. Indeed, it has been proposed that at nanoscale level spherical self-assemblies form the building blocks of zein particles with a structure similar to fractal-like aggregates [32]. According to the processing conditions, including mixing methods and ethanol/water ratio alongside the initial zein concentration, particle size from less than 100 to 500 nm are obtained [33, 34]. Ren et al. [35] reported an increase in particles size of zein microparticles from 504 to 1011 nm upon coacervation between zein and chitosan. Moreover, Wang et al. [34] obtained zein-chitosan microparticles of 6 µm by slowly pouring a zein solution into a chitosan solution while stirring at 5000 rpm and forming a suspension containing 7% of zein and 0.35% chitosan. Here a smaller particles size could have been anticipated due to the lower concentration of zein used in the formulation of the microparticles. However, a lower stir of 200 rpm was used which probably accounted for the relatively larger size of microparticles produced. Also, Fig. 2b seems to suggest that zein particles were coated with chitosan polymer as the micrograph showed a core shell-like structure for the particles formed without any active compounds. This result was confirmed with the FT-IR analysis, as discussed later. The coating of zein by chitosan would allow to overcome the main limitation of zein as encapsulating material since zein networks are brittle, thus prone to structural damage even at moderate levels of applied strain [36]. The result is particularly important for this study as the project aims to encapsulate bioactive compounds for food fortification and the encapsulated ingredient must resist stress conditions encountered during food processing. The suspension of the microparticles was dried using a freeze-dryer, oven-dryer and spray-dryer. As reported in Fig. 4, only the spray-drying process (Fig. 4a) allowed for the production of a free-flowing powder that would be more suitable for use as food fortification ingredient. The product from lyophilization was characterized by a flake-like structure with higher porosity (Fig. 4b) whereas a more compact film was obtained from the oven-dried sample (Fig. 4c). These results could be attributed to free chitosan molecules in the suspension that formed a network of polymer film in which the microparticles were embedded as a result of the solvent evaporation by freeze-drying or oven-drying. It is reasonable to hypothesise that, when zein particles were formed in the chitosan solution, some chitosan molecules interacted with zein particle surface via hydrophobic interaction, hydrogen bonding, Van der Waals forces and nonspecific electrostatic neutralisation of the opposite charges carried by the two biopolymers [37, 38]. Chitosan has positively charged ammonium groups (NH_3_^+^) at acidic pH whereas part of zein, due to the abundance of glutamic and aspartic acid with carboxylic anion (COO^−^), at pH above the isoelectric point (PI, about 3) is negatively charged [39]. The remaining chitosan in the solution is usually eliminated via centrifugation [38, 40] or filtration before spray-drying [32]. In this study, this separation step was excluded in the process to simplify and reduce the overall cost of the production, based on the principle that a droplet of free chitosan around the particles will be formed upon atomisation of the suspension. The resulting spray of droplets in hot gas current would instantaneously dry and form a powder of chitosan-zein particles embedded in a chitosan matrix (Fig. 5). Figure 6 shows the SEM images of empty chitosan-zein microparticles (CZ, Fig. 6a), chitosan-zein microparticles loaded with rutin (CZ-R, Fig. 6b) or rutin and vitamin D_3_ (CZ-VDR, Fig. 6c) after spray drying. The morphology and shape of empty and chitosan-zein loaded microparticles were similar, with spherical shape and wrinkled surface. This result indicates that the two bioactive compounds were effectively entrapped within the polymeric matrix with no effect on these physical proprieties of the produced microparticles. A liquid driven sol‐gel transition by diffusion transport mechanism across the interface with self‐organised surface deformation of chitosan hydrogels with wrinkle surface has been reported [41]. Here it is suggested that a rapid diffusion and evaporation of ethanol alongside water may have triggered a localized self-association of chitosan at the surface of the droplet since chitosan is insoluble in ethanol. The suspension contained about 20% ethanol. To summarize, the produced co-encapsulated vitamin D and rutin with spherical shape and particles size below 10 µm fulfill the industrial requirement to be used as ingredients for the fortification of food products such as chocolate, where a sandy or gritty mouthfeel is perceived when particles are larger than 25–35 μm [42].

### Encapsulation yield and efficiency, and loading capacity

Results of the main characteristics of the microparticles produced by spray-drying are presented in Table 1. Interestingly, the encapsulation yield was between 77 and 80% and there was no significant difference between the empty chitosan-zein and chitosan-zein loaded microparticles. This production yield was within the suggested 70% or more for a spray-dried powder to be sufficient to make the process economically feasible [43]. It should be noted that selected drying conditions were based on previous screening and that the good yield could be explained by the relatively low viscosity of the sample, the formation of smaller particles and the presence of ethanol, which led to rapid drying, and lower sickness of the particles on the wall of drying chamber. The encapsulation efficiency of rutin was 59% when used as single-core and decreased to 44% when co-encapsulated with vitamin D_3_. These values are close to the EE of 51% reported for resveratrol encapsulation inside zein and chitosan shell without any drying step [40], but definitely lower than the assessed encapsulation efficiency for vitamin D_3_ (75%). This difference could be attributed to the high vitamin D_3_ hydrophobicity when compared to rutin, resulting in a higher tendency of rutin to migrate in the antisolvent during the encapsulation process. Moreover, the difference in molecular size could have played a role, since the rutin molecular weight is 610.5 g/mol, almost twice that of vitamin D_3_ (384.6 g/mol). Vitamin D_3_ encapsulation efficiency was similar to the lower end value (76%) obtained for alpha-tocopherol encapsulated with zein and chitosan by antisolvent precipitation, followed by centrifugation and freeze-drying [37]. The amount of loaded vitamin D_3_ per unit weight, that is the loading capacity (LC), was 3.13% in the sample CZ-VDR and within the range (1.7–3.9%) reported by Luo et al. [43] when encapsulated vitamin D_3_ in zein protein and carboxymethyl chitosan shell. As explained before, a slight but significant (p < 0.05) reduction in rutin LC was observed during the co-encapsulation with vitamin D_3_ with LC assessed values of 1.69 and 1.23% for the samples CZ and CZ-VDR, respectively, representing a LC reduction of about 27%.

**Table 1:** Encapsulation yield and efficiency, loading capacity, powder a_w_ and moisture content of chitosan zein microparticles loaded with bioactive compounds or unloaded

Samples	Loading capacity (%)		Encapsulation efficiency (%)		Encapsulation yield (%)	Powder characteristics	
	Vitamin D_3_	Rutin	Vitamin D_3_	Rutin		a_w_	Moisture content (%)
CZ-VDR	3.13 ± 0.16	1.23 ± 0.04^b^	74.97 ± 3.77	44.36 ± 1.65^b^	77.7	0.296 ± 0.020^a^	3.33 ± 0.11^a^
CZ-R	–	1.69 ± 0.02^a^	–	59.20 ± 2.13^a^	78.4	0.280 ± 0.015^a^	3.32 ± 0.10^a^
CZ	–	–	–	–	80.1	0.285 ± 0.015^a^	3.27 ± 0.12^a^

*CZ-VDR* chitosan zein microparticles loaded with vitamin D_3_ and rutin, *CZ-R* chitosan-zein microparticles loaded with rutin, *CZ* chitosan zein microparticles with no active compound

Values with different superscripts within the same column are significantly different for p* <* 0.05

### Powder’s moisture content and water activity

Moisture content and water activity are two interrelated variables that help to predict powder ingredients stability and flowability since the latter also depends on the amount of water held by the powder particles [45] and has direct implication on the manufacturing efficiency and the uniformity of the resulting products. The moisture content of the obtained powders was about 3.3% while the assessed a_w_ values ranged between 0.285 and 0.296 (Table 1). There were no significant differences (p < 0.05) among the empty microparticles and those loaded with vitamin D_3_ and rutin (CZ-VDR) and only with rutin (CZ-R) for the two parameters. These values suggested that the spray-dried powders produced were microbiologically and biochemically stable, even if the low aw values indicated a high hygroscopicity of the samples, which should then be taken into account during storage at industrial level in order to eventually avoid caking [46, 47].

### X-ray powder diffraction and IR spectroscopy

**Fig. 7:**
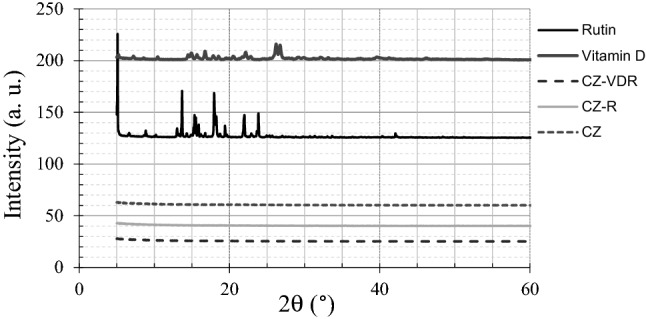
XRD patterns of pure bioactive compounds (rutin and vitamin D_3_) and chitosan zein microparticles loaded with vitamin D_3_ and rutin (CZ-VDR), with rutin (CZ-R) and unloaded (CZ)

**Fig. 8:**
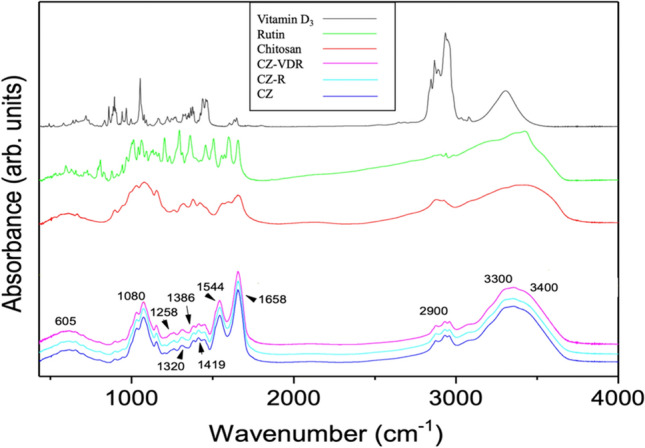
FT-IR spectra (range 4000–400 cm^-1^) of vitamin D_3_, rutin, chitosan and chitosan zein microparticles loaded with vitamin D_3_ and rutin (CZ-VDR), with rutin (CZ-R) and unloaded (CZ)

The X-ray diffraction technique was used to determine whether vitamin D_3_ and rutin were entrapped in the microparticles in a crystalline or amorphous state. The X-ray powder diffraction (XRPD) profiles of rutin, vitamin D_3_ and spray-dried microparticles are shown in Fig. 7. The profiles indicate that solid vitamin D_3_ and rutin were crystalline before encapsulation and spray-drying, as well-defined peaks were observed. Indeed, sharp diffraction peaks of pure rutin at *2θ* of 5.12°, 13.7°, 18.11°, 22.01° and 23.87° and those of vitamin D_3_, 26.72° and 26.81° are comparable to previously peaks reported by Hasanvand et al. [48] and Remanan and Zhu [49], respectively. Conversely, no peaks were detected in the powder of empty microparticles (CZ) or microparticles loaded with rutin (CZ-R) or rutin and vitamin D_3_ (CZ-VDR), indicating that the powders were amorphous. This is another advantage of the produced encapsulated vitamin D_3_ and rutin system as amorphous bioactive compounds generally have a better bioavailability than crystalline ones [50]. FT-IR was used to assess the nature of the interactions and the functional groups present at the surface of the microparticles. FT-IR spectra of chitosan and zein, the outer shell materials, non-encapsulated rutin and vitamin D_3_ alongside microparticles loaded with or without any active compounds are presented in Fig. 8, and the potential peaks in Table 2. FT-IR spectrum of pure chitosan exhibited a broad peak between 3400 and 3500 cm^−1^, which was assigned to the stretching vibration of N–H and O–H bond, respectively. Peak at 2900 cm^−1^ was due to the C-H stretch vibrations. The spectrum of empty (CZ) or loaded microparticles (CZ-VDR and CZ-R) is practically the same, indicating a good encapsulation of vitamin D_3_ and rutin. In the microcapsules spectra an interesting characterization peak was in the range of 3100 − 3500 cm^−1^, indicating the hydrogen bonding. As reported by other authors, the interaction of chitosan with proteins also occurs through hydrogen bonding. The hydrogen bonds are formed due to the interaction among the amino groups present in the protein structure and the amino groups in the chitosan chains [51, 52]. No shift was apparent for all microparticles in the vibration peaks of 1600 − 1700 cm^−1^ and 1530 − 1550 cm^−1^ corresponding to amide I and II bonds, respectively. The vibration peak at 1419 cm^−1^ in chitosan spectrum could be assigned to the symmetric stretching vibrations of carboxyl groups and it is present both in empty (CZ) or loaded microparticles (CZ-VDR-CZ-R) [53]. Other characteristic peaks of the microparticles are amide III band at 1320 cm^−1^, the area 900–1128 cm^−1^characteristic of pyranose units of the polysaccharide and NH_2_ bending vibrations at 605 cm^−1^ [54].

**Table 2:** FT-IR spectra showing observed peaks and probable functional groups

Wavenumber (cm^−1^)	Functional group	Reference
3100 − 3500	Stretching vibrational O–H	[55]
3310	Stretching vibration of N–H	[44]
3200–3450	N–H stretching	[55]
2928–2876	CH stretching	[54]
1658	C = O from amide groups and to NH_2_ deformation	[56, 57]
1600–1700	Amide I (C–O and C–N stretching)	[44, 57]
1530–1550	N–H deformation and C–N stretching (amide II)	[58]
1419	Symmetric stretching vibrations of carboxyl groups (CH-OH)	[54]
1386	CH bending	[54]
1320	Amide III band	[55]
1258	Aromatic ether ring vibrations (δ (O–H))	[55]
1080	C–O vibrational stretching (characteristics of primary alcoholic groups on chitosan structure)	[52]
1080	Vibration of pyranose units	[45]
900–1128	Pyranose units of the polysaccharide	[59, 60]
605	NH_2_ bending vibrations	[54]

## Conclusion

Vitamin D_3_ and rutin have been co-encapsulated in chitosan-zein microparticles via anti-solvent precipitation by continuous addition of an aqueous-ethanol solution of zein, containing the active compounds, into an aqueous chitosan solution (acting as anti-solvent), followed by spray drying. This was successfully achieved by utilising molecular interactions between chitosan and zein particles surfaces, through hydrophobic interaction, hydrogen bonding, Van der Waals forces and nonspecific electrostatic neutralisation of the opposite charges carried by the two biopolymers. Moreover, the ability of the spray drying process to generate droplets of suspension of zein particles in chitosan, which, in contact with hot gas current instantaneously dried to form a powder of chitosan-zein particles embedded in a chitosan matrix, contribute to the success microencapsulation process. The developed process eliminated the need for separating the chitosan which has not interacted with the zein core based on the principle that a droplet of free chitosan around the particles will be formed upon atomization of the suspension. The reported preliminary results indicated the potential use of the obtained free-flowing powders in food fortification since the size of the microparticles was below 10 microns. It is generally recognised that the human tongue can detect only particles larger than 30 μm, and therefore, the obtained microparticles are unlikely to negatively impact the fortified food mouthfeel. In addition, the XRPD profiles revealed the amorphous nature of the produced powders of microparticles loaded with rutin or rutin and vitamin D_3_ which would present a better bioavailability when compared to non-encapsulated crystalline form. The results of this study suggest that with a relatively simple process it is possible to co-encapsulate vitamin D_3_ with a molecule, such as rutin, that could potentially enhance its stability and activity, thus obtaining powders that could be used as ingredients for food fortification with vitamin D_3_. Taking all together, the obtained results are encouraging especially when considering the scale-up process for mass production. Indeed, the present work shows that a relatively simple process can be used to co-encapsulate vitamin D_3_ with a molecule, such as rutin, reducing the overall cost of the production and allowing the process cost-management. However, before being able to carry out the process on an industrial level it would be necessary to evaluate the microcapsules stability over time as well as the bioavailability of the co-encapsulated compounds in the resulting fortified food matrices Similarly, it would be appropriate to study the bioactivity of both the actives encapsulated alongside the correlated biometabolites.

## Data Availability

The data that support the findings of this study are available from the corresponding author, [RT], upon reasonable request.

## Abbreviations

*2θ*: Angle between reflected beam and reflected beam
Å: Ångstroms
CZ-R: Zein protein and chitosan microcapsules containing rutin
CZ-VDR: Zein protein and chitosan microcapsules containing vitamin D3 and rutin
CZ: Zein protein and chitosan empty microcapsules
DRV: Dietary reference value
EE: Encapsulation efficiency
EY: Encapsulation yield
FT-IR: IR spectroscopy measurement
LC: Loading capacity
MIR: Mid-infrared
XRPD: X-ray powder diffraction
